# Anticancer Therapy-Induced Alterations in Tumor-Derived Extracellular Vesicles: Molecular Mechanisms and Biological Effects

**DOI:** 10.3390/molecules31213901

**Published:** 2026-10-29

**Authors:** Mi Eun Kim, Jun Sik Lee

**Affiliations:** Department of Biological Science, Immunology Research Lab & BK21-Four Educational Research Group for Age-Associated Disorder Control Technology, Chosun University, Gwangju 61452, Republic of Korea; kimme@chosun.ac.kr

**Keywords:** tumor-derived extracellular vesicles, anticancer therapy, EV cargo, therapeutic resistance

## Abstract

Anticancer treatment alters the secretion and molecular composition of tumor-derived extracellular vesicles (EVs). Chemotherapy, ionizing radiation, and molecularly targeted therapy are associated with changes in EV-associated microRNAs (miRNAs), circular RNAs (circRNAs), long non-coding RNAs (lncRNAs), small nuclear RNAs (snRNAs), proteins, metabolic components, and tumor-associated antigens. Some treatment-associated EV changes are supported by molecular characterization alone, whereas others have been evaluated for functional effects in recipient cells. At the donor-cell level, altered EV release and cargo loading involve RAB family small GTPases, lysosomal degradation, multivesicular body–plasma membrane fusion mediated by soluble N-ethylmaleimide-sensitive factor attachment protein receptor (SNARE) proteins, the exocyst component Exo70, and RNA- and protein-loading processes. In recipient tumor cells, reported responses include changes in WNT/β-catenin and NOTCH signaling, phosphoinositide 3-kinase/protein kinase B (PI3K/AKT), mitogen-activated protein kinase (MAPK), platelet-derived growth factor receptor α (PDGFRα) signaling, glutamine metabolism, and oxidative responses. Evidence supporting individual cargo–response relationships includes both association-based observations and perturbation-based functional validation. In immune cells, C-X-C motif chemokine ligand 1 (CXCL1)-containing EVs increase programmed death-ligand 1 (PD-L1) expression in macrophages, and PD-L1-positive small extracellular vesicles (sEVs) reduce CD8^+^ T-cell proliferation and granzyme B production, whereas radiation-induced sEVs carrying tumor-associated antigens and heat shock proteins increase antigen presentation and CD4^+^ and CD8^+^ T-cell responses. This review separates treatment-associated molecular EV alterations from experimentally evaluated recipient-cell effects to clarify the evidence supporting therapy-induced EV responses.

## 1. Introduction

Extracellular vesicles (EVs) are membrane-enclosed particles released from cells and carry proteins, lipids, and nucleic acids derived from donor cells [1,2]. Tumor-derived EVs transfer molecular cargo to recipient cells and contribute to tumor growth, metastatic activity, immune regulation, and therapeutic resistance [1,3,4,5].

Anticancer treatment itself changes EV secretion and molecular cargo in tumor cells. However, EV-associated molecules identified in untreated tumors or drug-resistant cells are frequently discussed together with treatment-induced EV alterations. Detection of a resistance-associated RNA or protein in tumor-derived EVs does not establish that anticancer treatment changed its vesicular level or release. Distinguishing pre-existing tumor EV features from changes produced directly by therapy is therefore necessary for interpreting EV-associated treatment responses.

Previous reviews have examined tumor-derived EVs in cancer therapy and EV-mediated drug resistance [4,5]. The present review evaluates a sequence comprising anticancer treatment, intracellular changes in donor tumor cells, treatment-associated changes in EV secretion or molecular cargo, and molecular responses in recipient tumor or immune cells. This organization separates therapy-induced EV responses from EV features detected in untreated or drug-resistant tumor models. The objective of the present review is to evaluate tumor-derived EV alterations induced by chemotherapy, ionizing radiation, and molecularly targeted therapy, with emphasis on studies that directly associate treatment exposure with a measured EV alteration and a defined biological effect.

## 2. Anticancer Therapy-Induced Alterations in Tumor-Derived EVs

Anticancer treatment alters the release and molecular composition of tumor-derived EVs [6,7]. Changes in vesicle number and changes in EV-associated RNAs, proteins, and membrane molecules are considered separately because an increase in an EV-associated molecule does not by itself establish an increase in cargo per vesicle. The following sections examine alterations induced by chemotherapy, ionizing radiation, and molecularly targeted therapy. Comparison between studies requires attention to EV isolation and characterization procedures, normalization strategy, treatment dose and exposure time, and cell viability at EV collection. EV isolation and characterization affect the recovered vesicle populations and the amount of co-isolated non-vesicular material. Normalization to the number of recovered particles estimates EV-associated molecular abundance per measured particle, whereas normalization to viable donor-cell number or sample volume evaluates molecular output relative to the experimental input. An increase in total EV-associated RNA or protein without particle-normalized quantification does not establish increased cargo abundance per vesicle. Treatment dose and exposure time also affect EV release and cellular responses, and reduced cell viability complicates interpretation because treatment-related debris and non-vesicular material are potential contributors to extracellular preparations. Therapy-associated changes in EV-associated RNAs or proteins should therefore be interpreted together with EV yield, normalization strategy, treatment conditions, and viability data when these data are provided.

The interval between treatment exposure and EV collection is another important factor in cross-study interpretation. EVs collected during treatment or shortly after treatment exposure originate from tumor cells exposed to anticancer treatment, whereas EVs collected at later intervals originate from cells remaining after treatment. Drug resistance or cellular senescence in these surviving cells requires independent experimental verification. These collection intervals correspond to separate phases of the treatment response and should not be interpreted as equivalent. Reporting the interval between treatment exposure and EV collection is therefore important when evaluating EV release, EV-associated molecular levels, and recipient-cell effects between studies.

Clinical interpretation of circulating EV analyses requires additional considerations. Treatment-associated changes detected in circulating EVs do not by themselves establish a validated predictive biomarker. Blood contains EVs released from tumor cells and nonmalignant cell populations, making assignment of a circulating EV signal to tumor-derived EVs difficult. Clinical evaluation therefore requires evidence of tumor origin together with reproducible association between the EV alteration and treatment outcome.

Terminology in this review follows MISEV2023 recommendations [2]. EV is used as the general term when the subcellular origin of released vesicles has not been established. The terms exosome and sEV are retained when referring to vesicle preparations designated by these terms in the cited studies. Use of the term exosome in an individual study does not independently establish an endosomal origin.

### 2.1. Chemotherapy-Induced Alterations

In breast cancer, miRNA sequencing and RT-qPCR of serum exosomes collected before neoadjuvant chemotherapy and after one and four cycles of doxorubicin/paclitaxel identified increased miR-378a-3p and miR-378d after treatment [8]. In paclitaxel-treated breast cancer cells, RT-qPCR detected increased circular RNA derived from the BTB domain and CNC homolog 1 (circBACH1) in released exosomes [9].

Chemotherapy also modifies protein cargo and vesicle release. In triple-negative breast cancer cell cultures and orthotopic xenograft models, doxorubicin increased tumor-derived sEV secretion. Quantitative proteomic analysis also detected an increase in sEV-associated pentraxin 3 (PTX3), and genetic suppression of PTX3 or sEV secretion reduced pulmonary metastatic activity [10]. In ovarian cancer cells, cisplatin increased the release of EV-associated proteolytically active disintegrin and metalloprotease 17 (ADAM17). Patient-derived EVs induced amphiregulin (AREG) shedding and restored chemoresistance in ADAM17-deficient ovarian cancer cells, placing EV-associated ADAM17 upstream of epidermal growth factor receptor (EGFR) ligand release after chemotherapy [11].

Chemotherapy-induced EV alterations also include spliceosomal proteins and small nuclear RNAs. Proteomic analysis of paired ascitic fluids collected from patients with ovarian cancer before and after chemotherapy, together with in vitro secretome analysis of ovarian cancer cells, identified increased EV-associated small nuclear ribonucleoprotein 13 (SNU13) and synaptotagmin binding cytoplasmic RNA interacting protein (SYNCRIP) proteins and U12 and U6atac small nuclear RNAs after treatment [12].

### 2.2. Radiation-Induced Alterations

Ionizing radiation alters tumor-derived EV secretion and the levels of specific non-coding RNAs and proteins in released vesicles. In SW1783 and U-118MG glioblastoma cells, low-dose radiation increased exosome secretion. An increase in exosome-associated circ-METRN was also detected after irradiation. Serum exosome analysis during fractionated radiotherapy also detected treatment-associated circ-METRN changes in patients with glioblastoma [13]. Gamma irradiation also increased exosome release from MDA-MB-231 breast cancer and HeLa cervical cancer cells. Exosomes collected from irradiated cells increased ERK phosphorylation in recipient tumor cells, accompanied by increased proliferation and post-irradiation cell survival [14].

Radiation also modifies exosomal miRNA composition. In MIAPaCa-2 pancreatic cancer cells, miRNA microarray analysis identified radiation-associated changes in exosomal miRNAs, including miR-6823-5p [15]. In 4T1 and MDA-MB-231 breast cancer cells, irradiation increased exosome secretion. An increase in exosome-associated miR-21 was also detected after irradiation. Cell-based assays showed that exosomes released from irradiated 4T1 and MDA-MB-231 cells increased proliferation and migration of recipient breast cancer cells, while xenograft experiments showed increased tumor growth [16].

Protein cargo is also altered following irradiation. In oral squamous cell carcinoma cells, LC-MS/MS proteomic analysis detected increased sEV-associated levels of solute carrier family 1 member 5 (SLC1A5), a glutamine transporter, following irradiation [17].

### 2.3. Molecularly Targeted Therapy-Induced Alterations

EGFR-directed therapy alters both EV secretion and protein cargo in non-small cell lung cancer (NSCLC). In NSCLC cell models, nanoparticle tracking analysis showed that osimertinib increased exosome release from wild-type EGFR-expressing cells, whereas ELISA detected an increase in EV-associated wild-type EGFR [18]. In EGFR-mutant PC9 lung cancer cells, gefitinib reduced EV secretion and decreased EV-associated phosphorylated EGFR [19]. In EGFR-driven tumor cells, the second-generation EGFR kinase inhibitors CI-1033 and PF-00299804 induced rapid release of exosome-like EVs containing EGFR, phosphorylated EGFR, and genomic DNA. EGFR kinase inhibition also changed EV-associated phosphorylated EGFR at Tyr845, Tyr1068, and Tyr1173 together with ERK and AKT, while pharmacological inhibition of exosome formation or caspase activity reduced treatment-induced EV release [20]. EGFR-directed antibody treatment also changes EV protein cargo. In A-431 tumor cells, cetuximab reduced EV-associated EGFR and phosphorylated EGFR in association with the reduction observed in donor cells, and cetuximab itself was detected in the released EV fraction [21]. Single-EV analysis of EGFR-mutant NSCLC cells also showed treatment-associated alterations in EV surface proteins, including an increased programmed death-ligand 1 (PD-L1)/human epidermal growth factor receptor 2 (HER2)-positive EV population after osimertinib treatment [22]. EGFR-directed treatments produce inhibitor- and tumor-model-dependent changes in EV secretion [18,19]. HER2-directed antibody treatment also modifies tumor-derived EV protein composition. In ERBB2-positive breast cancer cells, trastuzumab induced a glycosylated 50-kDa isoform of the tetraspanin CD63 in released EVs and altered 51 EV-associated proteins, including DNA topoisomerase I (TOP1), as determined by high-resolution mass spectrometry [23].

Tyrosine kinase inhibitors targeting angiogenic signaling also modify EV production and molecular cargo. In 786-O and A498 renal cell carcinoma cells, nanoparticle tracking analysis showed increased small and large EV secretion after sunitinib or axitinib treatment. Quantitative proteomic analysis identified glycolytic proteins and glucose transporter 1 (GLUT1) in small extracellular vesicles (sEVs) released after treatment, and metabolic assays demonstrated increased glucose uptake and glycolytic activity in these vesicles [24]. In glioblastoma cells, bevacizumab was internalized through an early-endosomal route and subsequently detected on the surface of released EVs. Bevacizumab treatment also changed EV protein composition, whereas suppression of EV production increased the reduction in U87 glioblastoma cell viability produced by bevacizumab [25].

In RKO and HCT116 colorectal cancer cells, nanoparticle tracking analysis showed reduced exosome secretion after apatinib treatment. Reduced circulating exosome levels were also detected in apatinib-treated tumor-bearing mice [26]. Apatinib-mediated reduction in exosome secretion was accompanied by decreased exosomal PD-L1 [27].

Representative treatment-associated EV molecules, recipient-cell targets, and biological effects are summarized in Table 1.

Treatment-associated changes in EV release are described in Section 2 and Section 3. Increased EV-associated molecular abundance does not necessarily indicate increased cargo content per vesicle.

## 3. Molecular Mechanisms Responsible for Therapy-Induced EV Alterations

### 3.1. Regulation of EV Trafficking and Release

Anticancer treatment modifies EV release through molecular processes controlling endosomal trafficking, lysosomal degradation, and membrane fusion. Rab GTPases regulate intracellular vesicle trafficking associated with EV secretion. Cisplatin-resistant ovarian cancer cells exhibited increased RAB27A expression together with reduced RAB7 expression and lysosomal activity compared with cisplatin-sensitive cells. In the resistant cells, cisplatin exposure increased chemo-sEV secretion, whereas RAB27A silencing reduced chemo-sEV release [30]. Chemotherapy-responsive Rab proteins also regulate the export of drug-resistance proteins through EVs. In multidrug-resistant KBv200 cancer cells, transient vincristine exposure increased RAB8B expression and stimulated the secretion of EVs containing ATP-binding cassette subfamily B member 1 (ABCB1). RAB8B silencing reduced EV secretion and extracellular ABCB1, identifying RAB8B as a regulator of chemotherapy-induced ABCB1-containing EV release [31]. In chemoresistant colorectal cancer cells, RAB22A stabilized pyruvate kinase M2 (PKM2), promoted SNAP23 phosphorylation, and increased secretion of RAB22A- and PKM2-containing exosomes. Transfer of these exosomes induced acquired drug resistance in recipient colorectal cancer cells [32]. Targeted therapy also engages Rab-dependent regulation. Osimertinib increased RAB17 expression together with exosome release in NSCLC cells [18], whereas apatinib reduced RAB11 expression and exosome secretion in colorectal cancer cells [26].

Lysosomal degradation regulates the amount of vesicular material available for extracellular release. In cisplatin-resistant ovarian cancer cells, reduced lysosomal activity was associated with increased chemo-sEV secretion, whereas enhancement of lysosomal activity reduced vesicle release [30]. In colorectal cancer cells, apatinib promoted lysosomal-associated membrane protein 2 (LAMP2)-associated degradation of multivesicular bodies and reduced RAB11-dependent transport toward the plasma membrane, resulting in decreased exosome secretion [26]. MEK/ERK signaling also regulates EV release through lysosomal activity. In HT29 colorectal cancer cells, MEK inhibition with U0126 or trametinib reduced EV secretion and increased lysosomal acidification. MEK/ERK inhibition reduced c-MYC expression and induced nuclear translocation of MiT/TFE family transcription factors, including TFEB, TFE3, and microphthalmia-associated transcription factor (MITF), followed by increased expression of lysosomal genes such as ATP6V1B1. Activation of lysosomal degradation increased multivesicular body (MVB) degradation and reduced extracellular EV release [33].

Fusion of multivesicular bodies with the plasma membrane releases intraluminal vesicles as exosomes. In MCF-7 breast cancer cells, CRISPR-Cas9-mediated deletion of syntaxin-4, SNAP23, or VAMP7 reduced exosome secretion without altering MVB formation or intracellular transport. An in vitro membrane fusion assay confirmed that these three SNARE proteins form a functional complex responsible for MVB–plasma membrane fusion [34]. In A2780 and SKOV3 ovarian cancer cells, co-immunoprecipitation demonstrated that reduced O-GlcNAcylation of SNAP23 increased its interaction with VAMP8 and syntaxin-4. Nanoparticle tracking analysis and inductively coupled plasma mass spectrometry confirmed increased exosome secretion and exosomal cisplatin export following OGT suppression [35]. Apatinib reduces SNAP23 and VAMP2 together with exosome release in colorectal cancer cells [26].

Exo70, a component of the exocyst complex, is associated with vesicle docking and release at the plasma membrane. In A818-4 and PANC-1 pancreatic cancer cells, Exo70 knockdown or treatment with the Exo70 inhibitor Endosidin 2 (ES2) reduced exosome secretion and increased intracellular MVB accumulation. Exosome marker immunoblotting, transmission electron microscopy, and CD63 immunofluorescence were used to evaluate these alterations [36]. Apatinib also suppresses Exo70-dependent exosome secretion in colorectal cancer cells and decreases exosomal PD-L1 [27].

### 3.2. Molecular Regulation of Cargo Alterations

Therapy-associated increases in EV-associated molecules result from several processes, including increased intracellular expression, subcellular redistribution, increased EV-mediated export, and selective cargo loading. An increased EV-associated level alone does not establish selective loading. In CAL51 breast cancer cells, doxorubicin and paclitaxel increased EZH2 expression and signal transducer and activator of transcription 3 (STAT3) activity. Chromatin immunoprecipitation confirmed STAT3 binding to the miR-378a-3p and miR-378d promoters, whereas EZH2 or STAT3 silencing reduced the abundance of these miRNAs in both tumor cells and released exosomes. In BRAFV600-mutant melanoma, vemurafenib increased MITF expression, which activated the TRPM1 locus and increased miR-211-5p in melanoma cells and released EVs; selective loading of miR-211-5p was not tested in this model. Inhibition of miR-211-5p reduced proliferation of vemurafenib-resistant melanoma cells [37].

RNA-binding proteins also regulate chemotherapy-induced RNA loading into tumor-derived exosomes. In gastric cancer cells, cisplatin and paclitaxel increased expression of ubiquitin-specific peptidase 7 (USP7) and heterogeneous nuclear ribonucleoprotein A1 (hnRNPA1) and increased exosomal levels of the ferroptosis-associated long non-coding RNA lnc-ENDOG-1:1 (lncFERO) without a corresponding increase in cellular lncFERO. Co-immunoprecipitation in gastric cancer cells identified an interaction between USP7 and hnRNPA1, whereas USP7 suppression increased hnRNPA1 ubiquitination. Silencing USP7 or hnRNPA1 reduced exosomal lncFERO abundance without altering cellular lncFERO expression [38]. RNA-binding proteins also regulate RNA cargo loading into tumor-derived EVs. In NSCLC cells, heterogeneous nuclear ribonucleoprotein A2/B1 (hnRNPA2B1) recognized an exosomal sorting motif (EXO motif) in miR-122-5p and promoted its loading into EVs [39]. Y-box binding protein 1 (YBX1) regulated human Y RNA 4 (hY4 RNA) fragment loading into lung cancer-derived EVs, while YBX1 methylation at lysine 264 (K264) affected its interaction with hY4 RNA fragments and their vesicular levels [40]. Neither study investigated whether anticancer treatment alters hnRNPA2B1- or YBX1-dependent RNA loading into EVs.

Chemotherapy also changes EV cargo through intracellular redistribution. In ovarian cancer, chemotherapy induced nuclear-to-cytoplasmic relocation of spliceosomal components, accompanied by increased EV-associated SNU13 and SYNCRIP proteins and U12 and U6atac small nuclear RNAs [12]. No increase in transcription was detected. These results support intracellular redistribution of pre-existing spliceosomal components and increased EV-associated levels but do not establish selective cargo loading. Radiation also regulates EV RNA cargo through altered miRNA processing. In esophageal squamous cell carcinoma cells, irradiation increased histone H3 lysine 4 dimethylation (H3K4me2) at the non-coding RNA activated by DNA damage (NORAD) locus and increased NORAD expression. NORAD bound pumilio RNA-binding family member 1 (PUM1) and interfered with pri-miR-199a1 processing, reducing mature miR-199a-5p available for exosomal transfer. NORAD suppression increased exosomal miR-199a-5p, which targeted endonuclease/exonuclease/phosphatase family domain containing 1 (EEPD1) in recipient tumor cells and reduced ATR serine/threonine kinase (ATR)/checkpoint kinase 1 (Chk1) signaling, resulting in increased radiosensitivity [41]. This mechanism involves altered miRNA processing and intracellular availability rather than selective EV loading. Targeted inhibition of mTORC1 also changes exosomal miRNA export. In NSCLC cells, everolimus promoted RAB27A- and RAB27B-dependent secretion of miR-7-5p-containing exosomes and reduced intracellular miR-7-5p. Intracellular loss of miR-7-5p increased MNK1 expression and eIF4E phosphorylation, whereas delivery of exosomal miR-7-5p suppressed the MNK/eIF4E pathway. RAB27A/B silencing reduced everolimus-induced exosomal miR-7-5p secretion [42]. These results support regulated EV-mediated export of miR-7-5p rather than selective loading.

**Figure 1:**
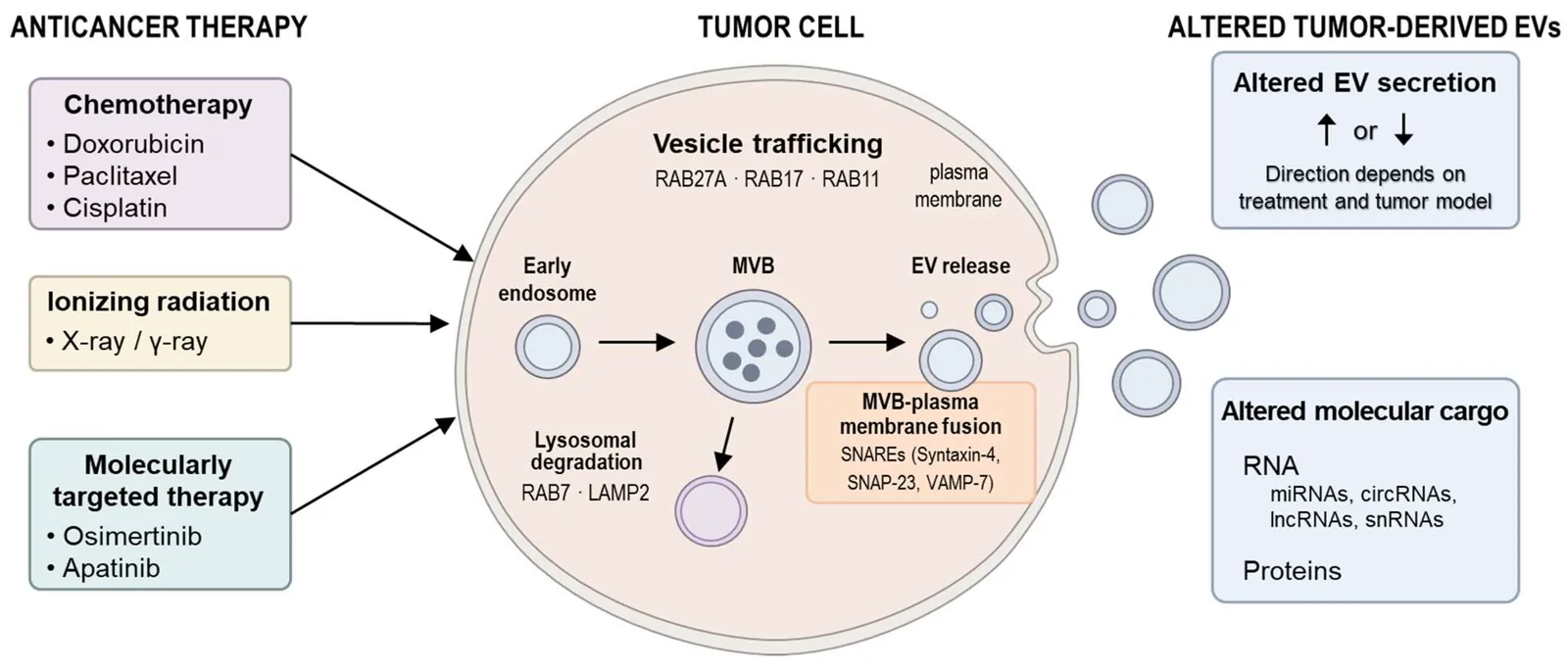
Molecular mechanisms of anticancer therapy-induced alterations in tumor-derived EVs. Chemotherapy, ionizing radiation, and molecularly targeted therapy induce changes in extracellular vesicle (EV) secretion and the levels of specific EV-associated miRNAs, circRNAs, lncRNAs, snRNAs, and proteins. RAB27A, RAB17, and RAB11 are involved in vesicle trafficking, whereas RAB7 and LAMP2 are associated with lysosomal degradation. The SNARE complex consisting of syntaxin-4, SNAP23, and VAMP7 mediates multivesicular body (MVB)–plasma membrane fusion during exosome secretion. The schematic integrates results obtained from separate cell-culture and animal models.

Selective protein loading was demonstrated for vascular endothelial growth factor (VEGF) during anti-VEGF treatment. In OVCAR5 and SKOV3 ovarian cancer cells, co-immunoprecipitation identified an interaction between CD63 and VEGF, whereas CD63 knockdown reduced sEV-associated VEGF. Bevacizumab-resistant ovarian cancer xenograft models exhibited increased serum sEV-associated VEGF, as determined by angiogenesis array analysis and immunoblotting. Vesicular VEGF remained inaccessible to anti-VEGF antibodies and activated VEGF signaling and tube formation in recipient endothelial cells despite bevacizumab treatment [43]. Therapy-associated changes in EV cargo therefore involve increased intracellular expression, subcellular redistribution, regulated EV-mediated export, or selective cargo loading, according to the evidence reported for each molecular cargo (Figure 1).

## 4. Biological Effects of Therapy-Altered Tumor-Derived EVs

### 4.1. Tumor Cell Responses

Mechanistic relationships are described when perturbation of EV cargo, molecular targets, or recipient-cell signaling altered the corresponding cellular response. When such functional validation was not reported, EV-associated molecules are described as being associated with the observed recipient-cell response. Therapy-altered tumor-derived EVs modify signaling pathways associated with the response of recipient tumor cells to anticancer treatment. Chemotherapy-induced exosomal miR-378a-3p and miR-378d suppressed Dickkopf WNT signaling pathway inhibitor 3 (DKK3) and NUMB endocytic adaptor protein (NUMB) expression in recipient breast cancer cells, activating WNT/β-catenin and NOTCH signaling. Increased β-catenin, cyclin D1, c-MYC, NOTCH1, HES1, and HEY1 accompanied increases in the CD44^+^/CD24^−^ cell population, mammosphere formation, and resistance to doxorubicin and paclitaxel [8]. Paclitaxel-induced exosomal circBACH1 bound miR-217 and relieved miR-217-mediated repression of G3BP stress granule assembly factor 2 (G3BP2). Reduction in circBACH1 in recipient breast cancer cells decreased cell viability, the CD44^+^/CD24^−^ population, mammosphere formation, and migration under paclitaxel treatment Conditioned medium from circBACH1-suppressed breast cancer cells also reduced tube formation by human umbilical vein endothelial cells (HUVECs) [9]. EV-associated long intergenic non-coding RNA VLDLR (linc-VLDLR) also regulates tumor-cell responses to anticancer drugs. In hepatocellular carcinoma cells, sorafenib, camptothecin, and doxorubicin increased cellular and EV-associated linc-VLDLR. Transfer of these EVs increased linc-VLDLR and ATP-binding cassette subfamily G member 2 (ABCG2) expression in recipient tumor cells and reduced drug-induced cell death. Silencing linc-VLDLR reduced ABCG2 expression, whereas ectopic ABCG2 expression reduced the effect of linc-VLDLR depletion on sorafenib-induced cell death [44]. In glioblastoma cells, radiation-induced exosomal circ-METRN suppressed miR-4709-3p and increased growth factor receptor-bound protein 14 (GRB14) expression, resulting in increased platelet-derived growth factor receptor α (PDGFRα) signaling. Circ-METRN-containing exosomes increased phosphorylated histone H2AX (γ-H2AX), tumor growth, and radioresistance, whereas inhibition of GRB14 suppressed these effects; PDGFRα expression reversed the effect of GRB14 inhibition [13]. In esophageal squamous cell carcinoma, irradiation increased exosomal high mobility group box 1 (HMGB1). Uptake of HMGB1-containing exosomes activated PI3K/AKT/FOXO3A signaling in recipient tumor cells and altered cyclin B1, CDK1, Bax, and Bcl-2 expression, resulting in G2/M accumulation and reduced apoptosis. Exosomal HMGB1 altered γ-H2AX signals after irradiation and increased radioresistance, whereas HMGB1 depletion in exosomes released from irradiated cells reduced these effects [45]. Osimertinib increased RAB17-dependent exosome release from wild-type EGFR-expressing NSCLC cells. Recipient EGFR-mutant cells internalized these exosomes through clathrin-dependent endocytosis, and the transferred wild-type EGFR activated PI3K/AKT and mitogen-activated protein kinase (MAPK) signaling. Exosome depletion from conditioned medium or cetuximab-mediated blockade of exosomal wild-type EGFR attenuated the exosome-induced increase in cell survival during osimertinib treatment [18].

Therapy-induced senescence and EV-mediated transfer of senescence-associated molecules also alter recipient tumor-cell responses. In multiple myeloma, doxorubicin-treated tumor cells released exosomes containing miR-433. Transfer of miR-433-containing exosomes to recipient multiple myeloma cells was accompanied by reduced cyclin-dependent kinase 6 (CDK6) expression and cellular senescence [46]. The biological activity of EVs released from therapy-induced senescent colorectal cancer cells was examined using cell-based assays, tumor xenografts, and colorectal cancer tissues obtained before and after anticancer treatment. EVs released from therapy-induced senescent colorectal cancer cells were enriched in serpin family E member 1 (SERPINE1) protein. EV-associated SERPINE1 bound NF-κB p65 and promoted its nuclear translocation, activating NF-κB signaling and increasing proliferation and migration in recipient colorectal cancer cells. Pharmacological inhibition of SERPINE1 with tiplaxtinin attenuated the tumor-promoting effects of senescent tumor cell-derived EVs [28].

In pancreatic cancer, exosomes released after irradiation increased intracellular reactive oxygen species and DNA damage in recipient cells, resulting in increased sensitivity to subsequent irradiation. miR-6823-5p was identified as a candidate involved in superoxide dismutase 1 (SOD1) suppression and increased intracellular reactive oxygen species [15]. sEV preparations with increased SLC1A5 levels, isolated from irradiated oral squamous cell carcinoma cells, increased glutamine uptake, glutamine metabolism, and proliferation in recipient tumor cells. Pharmacological inhibition of SLC1A5 reduced the tumor-promoting effects of radiation-induced sEVs in cell-culture and xenograft experiments [17].

### 4.2. Immune Cell Responses

Therapy-altered tumor-derived EVs regulate macrophage activity after anticancer treatment. In triple-negative breast cancer, paclitaxel and doxorubicin induced the release of C-X-C motif chemokine ligand 1 (CXCL1)-containing EVs from tumor cells. Macrophage uptake of these EVs increased CD206 and PD-L1 expression. CXCL1 depletion or neutralization reduced EV-induced PD-L1 expression in macrophages, whereas knockdown of embryonic ectoderm development protein (EED) reduced CXCL1-induced PD-L1 promoter activity. In tumor-bearing mice, CXCL1 depletion or macrophage depletion reduced EV-induced tumor growth and lung metastasis [47]. In macrophage–4T1 co-culture experiments, treatment of macrophages with these EVs increased tumor-cell invasion and resistance to paclitaxel-induced apoptosis. CXCL1 depletion or neutralization reduced tumor-cell invasion, whereas PD-L1 blockade attenuated EV-induced tumor-cell proliferation and invasion.

RNA sequencing of EVs released from irradiated esophageal squamous cell carcinoma (ESCC) cells identified increased DYNLL1 antisense RNA 1 (DYNLL1-AS1), and its biological activity was examined in macrophage assays, irradiated xenograft models, and human tumor tissues. EV-derived DYNLL1-AS1 bound SEC22 homolog B, a vesicle trafficking protein (SEC22B), in recipient macrophages and increased SEC22B expression. SEC22B interacted with FOXP1, whereas SEC22B or FOXP1 depletion reduced DYNLL1-AS1-induced PD-L1 promoter activity [48]. Macrophages exposed to irradiated ESCC-derived EVs or DYNLL1-AS1-enriched EVs reduced the proliferation of co-cultured CD3^+^ T cells and decreased IFN-γ and granzyme B concentrations in co-culture supernatants. Cisplatin-induced EVs also regulate CD4^+^ T-cell differentiation. In ovarian cancer cells, cisplatin increased EV secretion and enriched miR-181a-5p in tumor-derived EVs. EV-derived miR-181a-5p suppressed sirtuin 1 (SIRT1) in CD4^+^ T cells, increased FOXP3, and increased differentiation of CD25^+^ FOXP3^+^ regulatory T cells. Inhibition of miR-181a-5p reduced CD25^+^ FOXP3^+^ regulatory T-cell differentiation and reduced ovarian tumor growth in vivo [49].

In EGFR-mutant NSCLC cells, gefitinib increased PD-L1-positive sEV secretion through ALG-2-interacting protein X (ALIX). ALIX suppression reduced tyrosine kinase inhibitor (TKI)-induced PD-L1-positive sEV secretion. Exposure to these vesicles reduced Ki67 and granzyme B in CD8^+^ T cells, and paired peripheral blood obtained before and after EGFR-TKI treatment showed increased circulating sEV-associated PD-L1 after treatment [50]. Radiotherapy also changes tumor-derived exosomal miRNAs that regulate natural killer (NK) cells. In irradiated U87 glioblastoma cells, promoter methylation analysis identified TET methylcytosine dioxygenase 2 (TET2)-dependent demethylation of the miR-378 promoter, accompanied by increased miR-378a-3p expression and exosomal abundance. NK cells treated with exosomal miRNAs isolated from irradiated U87 cells exhibited reduced granzyme B secretion, as measured by ELISA. In PC3 prostate cancer xenograft mice and patients receiving radiotherapy, circulating exosomal miR-378a-3p abundance showed an inverse association with granzyme B [51]. DNA-containing tumor-derived exosomes also activate dendritic-cell innate immune signaling after anticancer treatment. Topotecan-treated breast cancer cells released DNA-containing exosomes that activated stimulator of interferon genes (STING) signaling and cytokine production in dendritic cells. In tumor-bearing mice, topotecan-induced tumor suppression was accompanied by activated dendritic-cell and CD8^+^ T-cell infiltration and was lost in STING-deficient mice [52]. Irradiated breast cancer cells released exosomes carrying three prime repair exonuclease 1 (TREX1)-sensitive double-stranded DNA, which increased CD40, CD80, and CD86 and activated STING-dependent type I interferon signaling in dendritic cells. These radiation-induced exosomes induced tumor-specific CD8^+^ T-cell responses in vivo [53].

**Figure 2:**
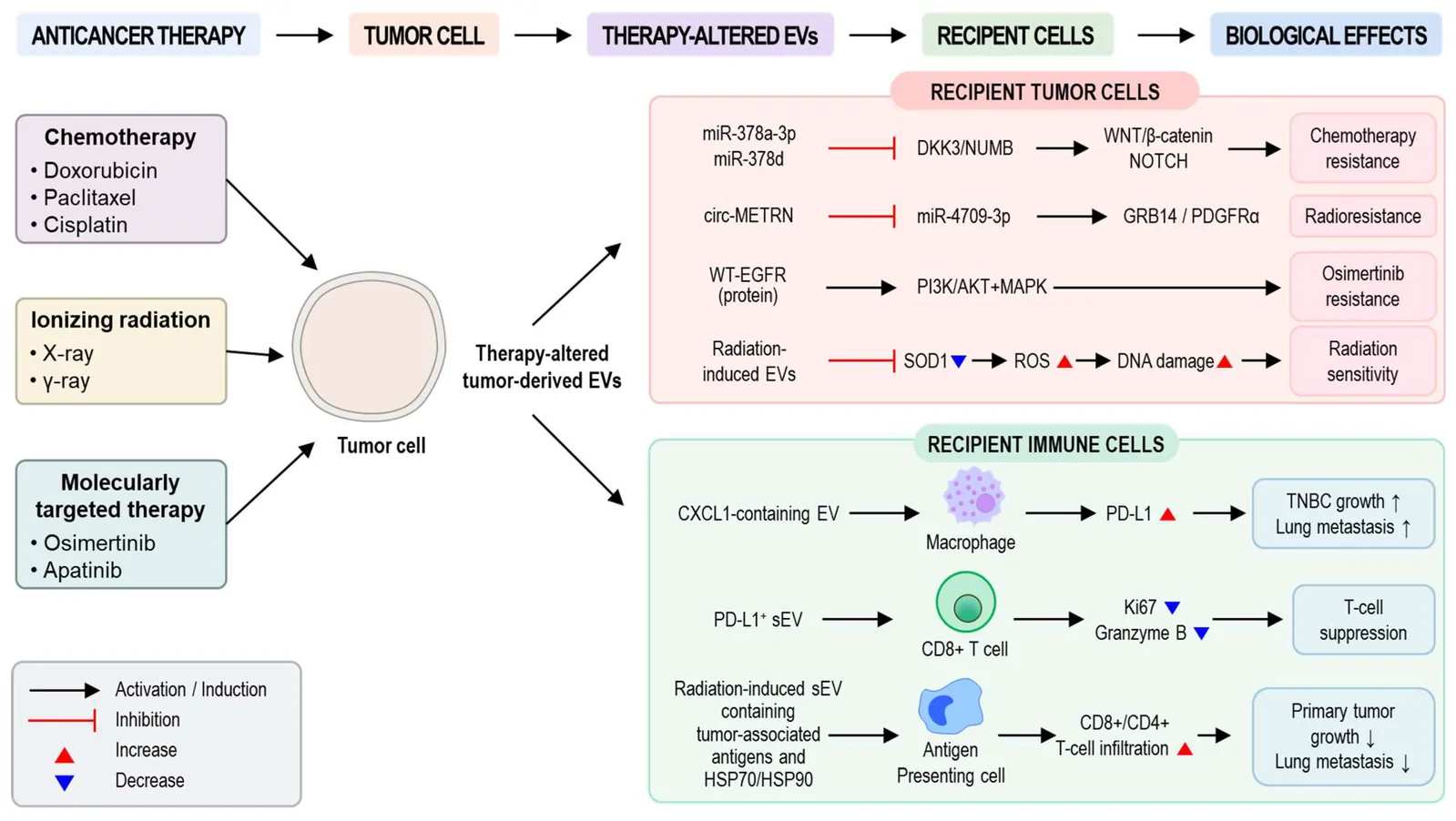
Biological effects of therapy-altered tumor-derived extracellular vesicles on recipient tumor and immune cells. Anticancer treatment changes the molecular cargo and biological activity of tumor-derived extracellular vesicles (EVs). In recipient breast cancer cells, chemotherapy-induced exosomal miR-378a-3p and miR-378d suppress DKK3 and NUMB, activating WNT/β-catenin and NOTCH signaling and promoting chemotherapy resistance. Radiation-induced exosomal circ-METRN suppresses miR-4709-3p, increasing GRB14 and PDGFRα expression and promoting radioresistance in glioblastoma cells. Exosomal wild-type EGFR activates PI3K/AKT and MAPK signaling in EGFR-mutated NSCLC cells, inducing osimertinib resistance. Exosomes released after irradiation increase ROS production and DNA damage in recipient tumor cells, with miR-6823-5p identified as a candidate involved in SOD1 suppression. In recipient immune cells, chemotherapy-induced CXCL1-containing EVs increase macrophage PD-L1 expression and promote triple-negative breast cancer growth and lung metastasis in mouse models. EGFR-TKI-induced PD-L1-positive small EVs (sEVs) reduce Ki67 and granzyme B expression in CD8^+^ T cells. Immunization with radiation-induced sEVs containing tumor-associated antigens and HSP70/HSP90 increases CD4^+^ and CD8^+^ T-cell infiltration and reduces primary tumor growth and experimental lung metastasis in mice. The schematic integrates results obtained from separate cell-culture, animal, and patient-derived studies. NSCLC, non-small cell lung cancer; ROS, reactive oxygen species; HSP, heat shock protein; TNBC, triple-negative breast cancer.

Radiation-induced sEVs also deliver tumor-associated antigens and heat shock proteins to immune cells. Radiation-induced sEV activity was examined using irradiated H22 and 4T1 tumor cells together with primary-tumor and lung-metastasis mouse models. Irradiated tumor cells released sEVs enriched in tumor-associated antigens together with heat shock protein (HSP)70 and HSP90. Radiation-induced sEVs were taken up by dendritic cells and increased CD8^+^ and CD4^+^ T-cell infiltration in tumor-bearing mice. CUB domain-containing protein 1 (CDCP1) was identified as a tumor-associated antigen enriched in radiation-induced sEVs. Immunization with CDCP1-derived peptides increased CD8^+^IFN-γ^+^ T cells and reduced primary and metastatic 4T1 tumor growth [29]. Taken together, irradiation-associated EVs produced both tumor-promoting and radiosensitizing responses in the cited experimental models. Circ-METRN-containing exosomes from low-dose-irradiated glioblastoma cells and HMGB1-containing exosomes from irradiated esophageal squamous cell carcinoma cells increased radioresistance, with molecular perturbation experiments supporting the involvement of circ-METRN and HMGB1 [13,45]. Exosomes collected 48 h after 4-Gy γ-irradiation of MDA-MB-231 and HeLa cells increased recipient-cell proliferation, ERK phosphorylation, and clonogenic survival following irradiation [14]. Exosomes from 10-Gy-irradiated 4T1 breast cancer cells increased proliferation, migration, and survival after irradiation; although EV-associated miR-21 levels increased, its individual contribution was not established by selective cargo depletion [16]. Radiation-induced sEV preparations with increased SLC1A5 levels enhanced glutamine uptake and proliferation in oral squamous cell carcinoma cells, and SLC1A5 inhibition attenuated these effects [17]. Conversely, exosomes from 5-Gy-irradiated MIAPaCa-2 cells increased ROS accumulation, DNA damage, and radiosensitivity in recipient pancreatic cancer cells [15]. Radiation-induced sEVs also increased CD4^+^ and CD8^+^ T-cell infiltration in mouse tumor models [29], whereas exosomal miR-378a-3p was associated with reduced NK-cell granzyme B secretion [51]. The cited studies used varied irradiation doses, donor tumor models, EV collection intervals, and recipient-cell assays but did not directly evaluate the individual contribution of these experimental variables to the contrasting outcomes (Figure 2).

## 5. Conclusions

Anticancer treatment alters the release and molecular composition of tumor-derived EVs and changes the RNAs and proteins delivered to recipient tumor and immune cells. Current evidence supports several intracellular processes responsible for therapy-associated EV alterations, including Rab-dependent vesicle trafficking, lysosomal degradation, SNARE-mediated membrane fusion, altered RNA processing, intracellular redistribution, regulated EV-mediated export, and selective cargo loading. Therapy-altered EVs are associated with chemotherapy response, radioresponse, targeted-therapy response, tumor-cell proliferation, metabolic changes, and immune-cell responses.

Evidence supporting the molecular origin of individual EV cargo changes is cargo-specific. Selective loading is supported for specific cargo through RNA-binding proteins or tetraspanin-dependent processes, whereas other studies report increased EV-associated molecular levels without establishing whether the increase originates from intracellular expression, subcellular redistribution, increased EV release, or selective loading. Interpretation of each treatment-associated EV alteration should therefore follow the experimental evidence reported for that molecular cargo.

Treatment-associated changes detected in circulating EVs should not be regarded as validated predictive biomarkers without additional clinical evidence. Circulating EV populations contain vesicles derived from tumor and nonmalignant cells, and assignment of an EV alteration to tumor-derived vesicles remains difficult. Clinical studies should define the interval between treatment exposure and EV collection, establish the tumor origin of the EV population under analysis, and determine whether EV alterations are reproducibly associated with treatment outcome in patient-derived material.

## Figures and Tables

**Table 1:** Representative treatment-associated EV molecules, experimental models, recipient cells, molecular targets/pathways, and biological effects.

Cancer Type/Experimental Model	Treatment	EV Type	EV-Associated Molecule(s)	Recipient Cell	Molecular Target/Pathway	Biological Effect	Ref.
Breast cancer/ Patient serum; cell culture; xenograft	Paclitaxel or doxorubicin	Exosomes	miR-378a-3p ↑; miR-378d ↑	Breast cancer cells	DKK3 ↓/WNT/β-catenin; NUMB ↓/NOTCH	Doxorubicin/paclitaxel resistance; increased CD44^+^/CD24^−^ breast cancer cell population and mammosphere formation	[8]
Breast cancer/ Cell culture; orthotopic xenograft	Doxorubicin	sEVs	PTX3 ↑	—	PTX3-associated metastatic activity	Pulmonary metastasis	[10]
Breast cancer/Cell culture	Paclitaxel	Exosomes	circBACH1 ↑	Breast cancer cells	miR-217/G3BP2	Paclitaxel resistance; increased breast cancer cell viability, mammosphere formation, and migration	[9]
Ovarian cancer/Patient ascites; cell culture	Chemotherapy	EVs	SNU13, SYNCRIP, U12, and U6atac ↑	Ovarian cancer cells	SNU13/SYNCRIP-associated therapy resistance; U12/U6atac-associated tumor growth	Cisplatin resistance in recipient ovarian cancer cells; increased tumor growth associated with extracellular U12/U6atac snRNAs	[12]
Colorectal cancer/Cell culture; xenograft; patient tumor	Chemotherapy or irradiation	EVs	SERPINE1 ↑	Colorectal cancer cells	NF-κB p65 nuclear translocation	Increased recipient colorectal cancer-cell proliferation, migration, and invasion; increased tumor growth in xenograft models	[28]
Glioblastoma/Cell culture; patient serum	Low-dose radiation	Exosomes	circ-METRN ↑	Glioblastoma cells	miR-4709-3p/GRB14/PDGFRα	Increased glioblastoma-cell proliferation, migration, invasion, and radioresistance	[13]
Pancreatic cancer/Cell culture	Ionizing radiation	Exosomes	miR-6823-5p ↑	Pancreatic cancer cells	SOD1 ↓; ROS ↑ (miR-6823-5p-associated candidate mechanism)	DNA damage; radiation sensitivity	[15]
Oral squamous cell carcinoma/Cell culture; xenograft	Ionizing radiation	sEVs	SLC1A5 ↑	Oral squamous cell carcinoma cells	SLC1A5-dependent glutamine uptake and metabolism	Tumor-cell proliferation	[17]
Multiple tumor models/Cell culture; mouse tumor models	Ionizing radiation	sEVs	Tumor-associated antigens; HSP70 ↑; HSP90 ↑	Antigen-presenting cells; T cells	Tumor antigen presentation and T-cell activation	Increased CD4+ and CD8+ T-cell infiltration and reduced primary tumor growth and experimental lung metastasis following immunization with radiation-induced sEVs	[29]
Non-small cell lung cancer/Cell culture	Osimertinib	Exosomes	Wild-type EGFR ↑	EGFR-mutant NSCLC cells	PI3K/AKT and MAPK signaling	Osimertinib resistance	[18]

(↑, increased; ↓, decreased; —, not assessed).

## Data Availability

No new data were created or analyzed in this study. Data sharing is not applicable to this article.
